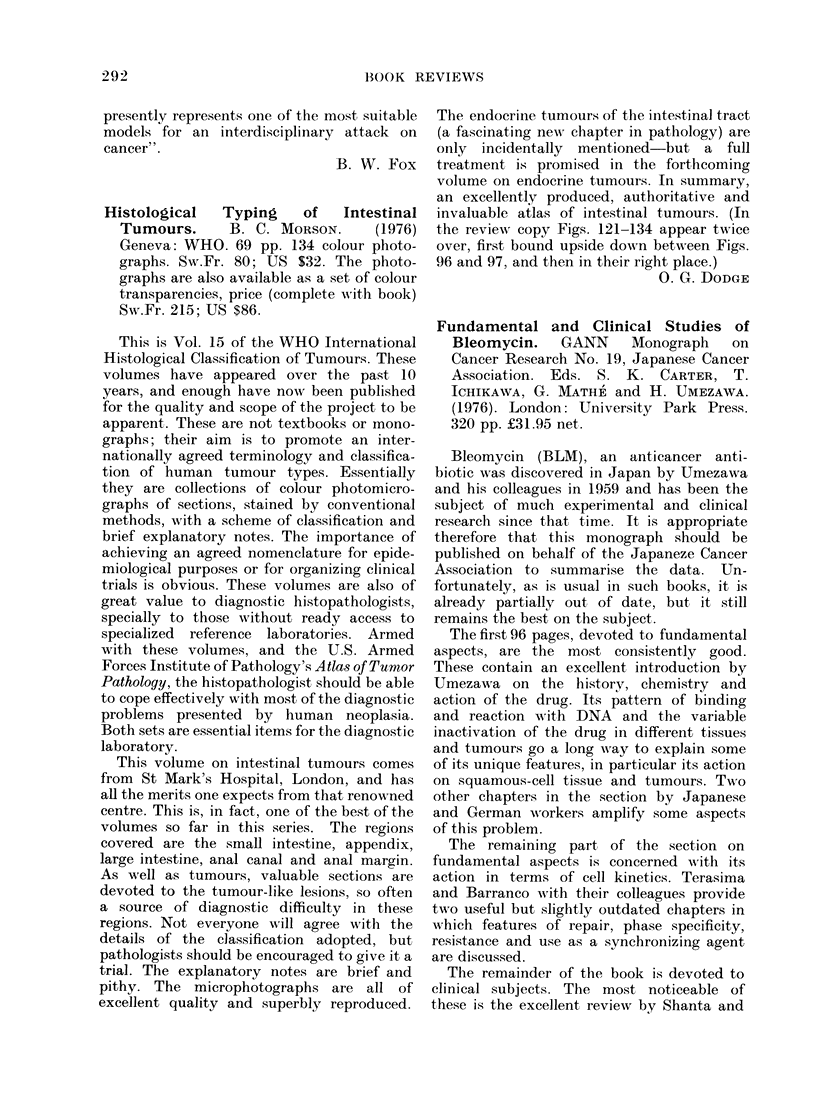# Histological Typing of Intestinal Tumours

**Published:** 1977-08

**Authors:** O. G. Dodge


**Histological Typing of Intestinal Tumours.** B. C. Morson. (1976) Geneva: WHO. 69 pp. 134 colour photographs. Sw.Fr. 80; US $32. The photographs are also available as a set of colour transparencies, price (complete with book) Sw.Fr. 215; US $86.

This is Vol. 15 of the WHO International Histological Classification of Tumours. These volumes have appeared over the past 10 years, and enough have now been published for the quality and scope of the project to be apparent. These are not textbooks or monographs; their aim is to promote an internationally agreed terminology and classification of human tumour types. Essentially they are collections of colour photomicrographs of sections, stained by conventional methods, with a scheme of classification and brief explanatory notes. The importance of achieving an agreed nomenclature for epidemiological purposes or for organizing clinical trials is obvious. These volumes are also of great value to diagnostic histopathologists, specially to those without ready access to specialized reference laboratories. Armed with these volumes, and the U.S. Armed Forces Institute of Pathology's *Atlas of Tumor Pathology*, the histopathologist should be able to cope effectively with most of the diagnostic problems presented by human neoplasia. Both sets are essential items for the diagnostic laboratory.

This volume on intestinal tumours comes from St Mark's Hospital, London, and has all the merits one expects from that renowned centre. This is, in fact, one of the best of the volumes so far in this series. The regions covered are the small intestine, appendix, large intestine, anal canal and anal margin. As well as tumours, valuable sections are devoted to the tumour-like lesions, so often a source of diagnostic difficulty in these regions. Not everyone will agree with the details of the classification adopted, but pathologists should be encouraged to give it a trial. The explanatory notes are brief and pithy. The microphotographs are all of excellent quality and superbly reproduced. The endocrine tumours of the intestinal tract (a fascinating new chapter in pathology) are only incidentally mentioned—but a full treatment is promised in the forthcoming volume on endocrine tumours. In summary, an excellently produced, authoritative and invaluable atlas of intestinal tumours. (In the review copy Figs. 121–134 appear twice over, first bound upside down between Figs. 96 and 97, and then in their right place.)

O. G. Dodge